# Interrupt or reinforce? The impact of timeout on momentum in basketball game

**DOI:** 10.3389/fpsyg.2025.1673186

**Published:** 2025-10-13

**Authors:** Mingjia Qiu, Kaimin Zhang, Yafei Chao, Mingxin Zhang

**Affiliations:** ^1^School of Athletic Performance, Shanghai University of Sport, Shanghai, China; ^2^Shanghai Key Lab of Human Performance, Shanghai University of Sport, Shanghai, China

**Keywords:** sports performance analysis, timeout effect, coaching decisions, situational factors, team sports

## Abstract

**Introduction:**

Timeouts are often viewed by coaches as an important means of intervening in a game, but their impact on the game’s momentum still lacks empirical research. This study aims to evaluate the impact of timeouts on momentum, defined as excellent offensive and defensive performance over a short period in basketball games, and to analyze the moderating role of contextual factors.

**Methods:**

A total of 4,051 timeouts from 1,235 elite professional basketball games were analyzed. Momentum was measured at short (48 s), medium (96 s), and long (144 s) intervals before and after each timeout. *T-*test (or Wilcoxon signed-rank test) were used to examine the difference in momentum before and after timeouts, and linear mixed models were employed to assess the impact of contextual factors.

**Results:**

Timeouts significantly increased team momentum, particularly when the team was in a disadvantaged status. Conversely, when the team was in an advantageous status, timeouts tended to reduce momentum. Contextual factors significantly moderated the effects of timeouts: better effects were observed during regular game periods, whereas timeouts were less effective in the last 5 min or against high-level opponents. Moreover, the medium- and long-term effects of timeout are more susceptible to contextual modulation, whereas short-term effects remained relatively stable.

**Discussion:**

Timeouts are an important intervention to influence momentum in basketball, and their effectiveness depends on game context and team status. These findings provide practical insights for coaches, suggesting that timeout strategies should be tailored to game conditions and situational dynamics to enhance their effectiveness.

## 1 Introduction

In team sports, coaches undertake multiple responsibilities during games, including tactical decisions, player rotation, timeout management, psychological support, and in-game adjustments to ensure optimal team performance and increase the likelihood of victory. Basketball is characterized by a fast pace, intense physical confrontations, and complex dynamics shaped by multiple factors. Coaches must continuously adapt to this dynamic, strategic, and tactical environment to make the best decisions for their teams (Clay and Clay, 2014). As a rule-governed in-game decision-making tool, timeouts are limited in number during games. Therefore, coaches must carefully weigh the pros and cons before using them. Timeouts in basketball may serve multiple purposes. Tactically, timeouts offer coaches an opportunity to reconfigure offensive or defensive strategies, particularly when the team is at a disadvantage, aiming to reverse momentum (Allgrunn et al., 2024; Andrews, 2015; Gibbs et al., 2022). Physiologically, timeouts facilitate the recovery of players’ physical energy and concentration, thereby enhancing subsequent performance. Psychologically, communication and instruction from coaches during timeouts help players remain calm and focused under pressure, minimizing performance fluctuations caused by stress or inattention (Abreu et al., 2017; Gutiérrez-Aguilar et al., 2016).

Typically, the outcome of the first possession after a timeout is regarded as the most direct indicator of timeout effectiveness. However, the influence of a timeout may extend over multiple possessions or serve a specific function, especially during close scores, momentum shifts, or decisive phases of the game. For instance, in critical moments, coaches may use timeouts to disrupt the opponent’s rhythm and interfere with their free-throw performance (Goldschmied et al., 2023). In fact, such contextual factors are often considered in contemporary performance analysis and are viewed as fundamental to understanding the game. Specifically, relevant studies often incorporate factors such as opponent quality, and game period into the analytical framework (Gómez et al., 2011; Sampaio et al., 2013). Existing evidence suggests that the effects of timeouts are not consistently uniform but exhibit significant context dependence. For example, some studies have found that teams in the lead are more likely to succeed following a timeout (Vázquez-Estévez et al., 2025), suggesting that timeouts may reinforce existing advantages. In addition, offensive efficiency following timeouts is generally higher in the final 5 min of a game than during regular phases (Gómez et al., 2011), highlighting the increased strategic value of timeouts in clutch moments. These findings indicate that timeouts are not isolated events, their effects are modulated by game context, necessitating the inclusion of situational variables as key explanatory factors.

Although the strategic value of timeouts has been recognized in research, significant ambiguity and methodological challenges remain regarding their quantification, with no standardized evaluation approach established. Early studies used direct comparisons of points scored and conceded, such as evaluating a team’s performance in several possessions following a called timeout (Gómez et al., 2011). Researchers also assessed net benefits by comparing post-timeout performance between teams that called timeouts and those that did not (Sampaio et al., 2013). Saavedra and colleagues proposed the “timeout factor,” defined as the difference between a team’s post-timeout points per possession and their season average, to reflect performance improvement (Saavedra et al., 2012). These studies adopted possessions as the analytical framework, aligning with the authentic logic of the game, as basketball is inherently a possession-based sport in which offense, defense, and tactical adjustments occur within each possession. More recent studies have gone beyond scoring changes to examine shifts in tactical execution and offensive patterns after timeouts, revealing their potential role in regulating team strategies (Vázquez-Estévez et al., 2025). Overall, these studies offer diverse approaches to quantifying timeout effects and provide coaches and analysts with insights into timeout strategies.

However, basketball games are often characterized by high randomness and nonlinearity (De Saá Guerra et al., 2013; Gabel and Redner, 2012). Although timeouts are typically viewed as tools for tactical adjustment, they may serve a deeper role in modulating and intervening in the game’s momentum. From this perspective, depicting performance changes over only a few possessions after a timeout is insufficient to capture shifts or continuities in the overall trend or rhythm of the game. Hastie noted that one of the coach’s responsibilities is to recognize and intervene in the team’s current state, when the game’s randomness transitions into a positive or negative momentum, the coach must manage the team accordingly to reverse disadvantageous trends or solidify existing advantages (Hastie, 1999). Therefore, the value of timeouts should not be limited to tactical adjustments or physical recovery, their core function may lie in disrupting the opponent’s positive momentum, halting the team’s decline, or initiating a new phase of advantage. In fact, some studies have provided empirical evidence supporting this view. Mace found in college basketball games that coaches often called timeouts when their team was being suppressed, which significantly reduced the opponent’s reinforcement rate (Mace et al., 1992). This finding was later validated by Roane in women’s college basketball (Roane et al., 2004). These small-sample studies indicate that timeouts reduce the opponent’s accumulated behavioral momentum. However, some studies have indicated that timeouts exert only minimal influence on altering game momentum and do not significantly enhance subsequent performance. Researchers have found no causal effect of timeouts on game performance, arguing that timeouts are often called during opponents’ scoring runs, and the apparent post-timeout improvement is actually attributable to the natural regression of scores to the mean (Permutt, 2011; Assis et al., 2021). Overall, while these studies highlight the importance of accounting for momentum fluctuations when analyzing timeout effects, their contradictory findings may stem from the high dependence of timeout effects on outcome measures and the analytical framework employed. Furthermore, to our knowledge, no study has investigated the scenario in which coaches call a timeout while their team is in a positive momentum state. This may be due to the rarity of such occurrences, as this behavior is often perceived as potentially disrupting the players’ current rhythm or undermining the team’s advantage (Allgrunn et al., 2024; Gibbs et al., 2022). Nonetheless, regardless of whether the decision is driven by tactical considerations, physical recovery, or rhythm control, evaluating timeouts under such circumstances can deepen our understanding of their strategic role.

The shift in a team’s performance over a period of time is commonly referred to as momentum in basketball, typically characterized by short-term dominance sustained over multiple possessions. Momentum is a complex, multilayered, and multidimensional phenomenon, with potential mechanisms involving psychological, physiological, and behavioral components (Taylor and Demick, 1994; Iso-Ahola and Blanchard, 1986; Iso-Ahola and Dotson, 2016). Given this, using momentum to analyze game dynamics and evaluate process-oriented interventions (e.g., timeouts) appears to have significant conceptual and practical value. However, the ambiguity of the concept has led to ongoing debate regarding whether momentum truly exists or is merely a cognitive illusion. Some researchers argue that momentum may merely reflect observers’ cognitive biases toward random events or retrospective rationalizations, or that it is a matter of statistical probability, thereby denying its real impact on game outcomes (Bar-Eli et al., 2006; O’Donoghue and Brown, 2009).

In response to this theoretical divergence, Qiu and colleagues recently proposed a momentum framework grounded in an operationalized definition. This framework integrates “time constraints” and “score differentials,” offering a practical empirical pathway for detecting and classifying momentum events in games (Qiu et al., 2024). The scientific validity of using score difference as a performance indicator in basketball analysis has long been recognized, as it can directly and stably reflect the relative superiority and inferiority of both sides, which is consistent with the logic of metrics such as offensive/defensive efficiency (Kubatko et al., 2007). At the same time, most advanced basketball metrics have adopted a “time-normalized” approach (such as per-minute statistics and plus–minus values expressed per minute), with the aim of correcting for time differences and enhancing comparability across players, teams, and situations. Therefore, operationalizing momentum as the rate of score difference change per unit time (which can be presented per minute) not only continues existing analytical practices but also enhances the intuitive interpretability of this metric among coaches and analysts. This method quantifies momentum as the ratio of net score difference to time, with a minimum trigger threshold defined as a net gain of +6 points within a 96-s time window. Qiu and colleagues found that momentum events meeting these criteria occurred significantly more often in winning teams and were moderated by game phase and opponent quality, demonstrating contextual dependency. Compared with previous momentum frameworks, Qiu’s framework demonstrates a better alignment with the study of timeout effects. The essence of the hot-hand effect lies in individual scoring continuity, making it more suitable for explaining players’ shooting choices or psychological states, yet insufficient to capture the overall offensive and defensive dynamics at the team level (Stone and Arkes, 2018; Pelechrinis and Winston, 2022; Morgulev and Avugos, 2023). The definition of scoring runs struggles to account for random fluctuations in games, and the absence of a unified threshold standard means that factors such as league type and team quality may substantially affect its applicability (Gibbs et al., 2022; Permutt, 2011). Although complex systems and nonlinear frameworks can portray the overall course of the game and provide new perspectives for understanding its complexity, they lack sufficient explanatory power in practical applications and cannot adequately match interventions at specific time points (Fewell et al., 2012; Luo et al., 2024). Qiu’s framework integrates contributions from both offense and defense, quantifies momentum changes, establishes a methodological foundation for incorporating momentum into empirical research and supports further investigation into how process-oriented interventions (e.g., timeouts) regulate momentum. Therefore, building on the framework proposed by Qiu, the present study systematically evaluates the impact of timeouts on momentum. The objectives are to: (1) assess momentum difference before and after timeouts, (2) examine whether timeout effectiveness differs under disadvantageous versus advantageous conditions, and (3) determine whether contextual variables moderate the effect of timeouts.

## 2 Materials and methods

### 2.1 Sample

The sample consisted of 1,235 regular-season games from the Chinese professional basketball league (CBA) across the 2021–2022, 2022–2023, and 2023–2024 seasons. Significant differences exist between the regular season and the playoffs in terms of game load, physical confrontation, and key performance indicators (García et al., 2013; Ferioli et al., 2021). Moreover, the regular season encompasses all teams, offering greater representativeness and consistency in format, whereas the playoffs involve only a subset of teams, and the particularities of context and tactical logic may compromise the generalizability of findings. For these reasons, only regular-season data were included in this study to ensure sample homogeneity and comparability. The data were obtained from the publicly available play-by-play records on the official CBA website, which have been validated for reliability and accuracy (Ou-Yang et al., 2025; Qiu et al., 2024). This study employed an observational design using publicly accessible data, without any direct contact or intervention with athletes or coaches. All data were anonymized prior to analysis to ensure that individual identities could not be traced. As this study did not involve experimental manipulation or personal data collection from human subjects, formal ethical approval was not required. However, all procedures adhered to the ethical guidelines of the authors’ institution.

In CBA, the timeout system follows the regulations of the International Basketball Federation (FIBA), with one additional modification. Each team may request a maximum of six timeouts per game: two long timeouts in the first half, three long timeouts in the second half, and one additional short timeout during the final 2 min of the game. A long timeout lasts 60 s, whereas a short timeout lasts 30 s. Importantly, unlike the NBA, the CBA does not employ official television timeouts. In this study, all analyses were based solely on team-requested timeouts.

### 2.2 Momentum

To assess differences in game momentum before and after timeouts, we adopted Qiu’s definition of momentum, which quantifies it as the ratio of score change to time elapsed (Qiu et al., 2024). Given that Qiu set the minimum threshold for momentum at 96 s, we calculated momentum for the 96 s preceding and following each timeout. In addition, prior studies (Lovato and Barreira, 2025; Mace et al., 1992; Roane et al., 2004; Sampaio et al., 2013) suggest that analyzing both short- and long-term changes post-timeout can provide meaningful insights into immediate and prolonged game dynamics. Therefore, momentum was also computed over shorter (48 s) and longer (144 s) windows to evaluate short- and long-term effects. Thus, the study defined three temporal windows: short-term (48 s), mid-term (96 s), and long-term (144 s). Timeouts that were too close to each other or followed by a period ending shortly afterward were excluded, as they interfered with the assessment of pre- and post-timeout momentum. A total of 4,051 timeout samples were included in the final analysis.

In addition to evaluating the overall effects of all timeout samples (*n* = 4,051), we further extracted “disadvantaged state” samples (*n* = 3,141) and “advantaged state” samples (*n* = 35) from the complete dataset to analyze the effects of timeouts under different game states. Specifically, when momentum values were negative across short-, mid-, and long-term windows before the timeout, the team was considered to be in a disadvantageous state. Conversely, if momentum was positive in all three pre-timeout windows, the team was deemed to be in an advantageous state. Consistency in momentum direction across the three windows reflected a sustained state of either advantage or disadvantage. We adopted this strict classification approach because momentum is conceptualized as a continuous psychological and behavioral trend rather than a transient performance fluctuation (Iso-Ahola and Dotson, 2014, 2016; Briki, 2017). Moreover, as basketball games are inherently nonlinear (Gabel and Redner, 2012; De Saá Guerra et al., 2013), the occurrence of consistent linear trends across multiple time scales suggests the emergence of special circumstances and phenomena, which better align with the definition of momentum. Therefore, this classification approach aimed to avoid biases arising from incidental fluctuations in game state.

### 2.3 Contextual factors

Contextual variables were included based on previous basketball performance analysis studies (Alonso-Pérez-Chao et al., 2024; Gómez et al., 2017; Sampaio et al., 2013), including game period (regular/last 5 min), opposition quality (low/medium/high), and score status (winning/balanced/losing). Among them, the classification of game period was based on readily available information. While opposition quality and score status were categorized using cluster analysis based on seasonal win rates and score differentials at the time of timeout, following previous studies (Alonso-Pérez-Chao et al., 2024; Gómez et al., 2017).

### 2.4 Statistical analysis

Data were presented using descriptive statistics. First, the Shapiro–Wilk test was employed to examine the normality of all variables. For variables with normal distributions, paired-sample t-tests were used to compare momentum before and after timeouts. For non-normally distributed variables, the Wilcoxon signed-rank test was applied. To evaluate the influence of contextual variables on timeout effectiveness, linear mixed models were constructed. Specifically, the difference in momentum before and after timeouts was set as the dependent variable, while game period (two levels), opposition quality (three levels), and score status (three levels) were treated as fixed effects, and team was included as a random effect to account for repeated measures. Present the estimated marginal means (EMM) and 95% confidence intervals (95%CI) to display the variables of the linear mixed model. Bonferroni corrections were applied to all multiple comparisons to control Type I error, and pairwise comparisons were conducted where appropriate. Cohen’s d was calculated to assess effect sizes (ES), with the following interpretations: <0.2 = Trivial; 0.20–0.59 = Small; 0.60–1.19 = Moderate; 1.2–1.99 = Large; ≥2.0 = Very Large (Batterham and Hopkins, 2006). Statistical significance was set at *p* < 0.05. All analyses were conducted using IBM SPSS software (Version 29; IBM Corp, Armonk, NY, USA).

## 3 Results

Table 1 presents the comparison of momentum before and after timeouts. Momentum significantly increased after timeouts across short-term (*p* < 0.001, Moderate), mid-term (*p* < 0.001, Moderate), and long-term (*p* < 0.001, Moderate) windows. Under disadvantage status, timeouts significantly increased momentum in the short-term (*p* < 0.001, Moderate), mid-term (*p* < 0.001, Small), and long-term (*p* < 0.001, Small) periods. In contrast, timeouts under advantage status significantly reduced team momentum in the short-term (*p* < 0.001, Moderate), mid-term (*p* = 0.005, Small), and long-term (*p* = 0.014, Small).

**Table 1:** Comparison of momentum before and after timeout in different status.

Status	Time windows	Momentum before timeout	Momentum after timeout	*p*	Effect size
All	Short-term	−0.072 ± 0.051	0.005 ± 0.055	<0.001*	Moderate
	Mid-term	−0.041 ± 0.035	0.000 ± 0.032	<0.001*	Moderate
	Long-term	−0.029 ± 0.026	−0.000 ± 0.035	<0.001*	Moderate
Disadvantage	Short-term	−0.085 ± 0.045	0.005 ± 0.056	<0.001*	Moderate
	Mid-term	−0.050 ± 0.027	0.000 ± 0.032	<0.001*	Small
	Long-term	−0.037 ± 0.021	−0.000 ± 0.026	<0.001*	Small
Advantage	Short-term	0.045 ± 0.025	−0.000 ± 0.051	<0.001*	Moderate
	Mid-term	0.024 ± 0.028	0.003 ± 0.030	0.005*	Small
	Long-term	0.017 ± 0.024	0.001 ± 0.028	0.014*	Small

*p < 0.05.

Tables 2–4 presents the influence of contextual variables on the effectiveness of timeouts. The short-term effect was influenced by game period (*p* < 0.001, Small). The mid-term effect was influenced by game period (*p* < 0.001, Small), and opposition quality (*p* = 0.002, Trivial). The long-term effect of timeouts was influenced by game period (*p* < 0.001, Trivial) and score status (*p* = 0.016, Small). Under disadvantage status, the short-term effect was influenced by opposition quality (*p* = 0.030, Trivial). The mid-term effect was not influenced by contextual factors. The long-term effect was influenced by opposition quality (*p* = 0.012, Trivial) and score status (*p* < 0.001, Trivial). Under advantage status, the short-term effect was influenced by opposition quality (*p* = 0.014, Moderate) and score status (*p* = 0.003, Moderate). The mid-term and long-term effect were not influenced by contextual factors.

**Table 2:** The impact of contextual factors on the effectiveness of timeout in all status.

Variables		Time windows					
		Short-term		Mid-term		Long-term	
		EMM [95%CI]	*p* (ES)	EMM [95%CI]	*p*	EMM [95%CI]	*p*
Game period	Regular	0.079 [0.076, 0.083]	<0.001* (small)	0.042 [0.040, 0.044]	<0.001* (small)	0.030 [0.028, 0.032]	<0.001* (trivial)
	Last 5 min	0.060 [0.051, 0.068]		0.032 [0.027, 0.038]		0.023 [0.018, 0.027]	
Opposition quality	High-quality	0.066 [0.061, 0.072]	0.133 (trivial)	0.035 [0.031, 0.039]	0.002* (trivial)	0.025 [0.022,0.027]	0.093 (trivial)
	Medium-quality	0.071 [0.066, 0.076]		0.041 [0.037, 0.044]		0.027 [0.025, 0.030]	
	Low-quality	0.071 [0.064, 0.079]		0.036 [0.031, 0.041]		0.026 [0.023, 0.030]	
Score status	Winning	0.069 [0.063, 0.075]	0.855 (trivial)	0.037 [0.033, 0.040]	0.695 (trivial)	0.025 [0.022, 0.028]	0.016* (trivial)
	Balanced	0.069 [0.063, 0.075]		0.037 [0.033, 0.041]		0.025 [0.022, 0.028]	
	Losing	0.071 [0.064, 0.077]		0.038 [0.034, 0.042]		0.029 [0.026,0.032]	

*p < 0.05.

**Table 3:** The impact of contextual factors on the effectiveness of timeout in disadvantage status.

Variables		Time windows					
		Short-term		Mid-term		Long-term	
		EMM [95%CI]	*p* (ES)	EMM [95%CI]	*p*	EMM [95%CI]	*p*
Game period	Regular	0.093 [0.090, 0.096]	0.062 (trivial)	0.051 [0.049, 0.053]	0.523 (trivial)	0.038 [0.036, 0.040]	0.281 (trivial)
	Last 5 min	0.083 [0.073, 0.093]		0.049 [0.043, 0.055]		0.035 [0.030, 0.040]	
Opposition quality	High-quality	0.083 [0.077, 0.089]	0.030* (trivial)	0.048 [0.045, 0.052]	0.182 (trivial)	0.034 [0.031, 0.037]	0.012* (trivial)
	Medium-quality	0.086 [0.081, 0.092]		0.051 [0.047, 0.054]		0.036 [0.033, 0.039]	
	Low-quality	0.094 [0.086, 0.103]		0.052 [0.047, 0.057]		0.040 [0.035, 0.044]	
Score status	Winning	0.087 [0.081, 0.094]	0.690 (trivial)	0.049 [0.045, 0.053]	0.340 (trivial)	0.035 [0.032, 0.039]	<0.001* (trivial)
	Balanced	0.087 [0.081, 0.093]		0.050 [0.046, 0.054]		0.034 [0.031, 0.037]	
	Losing	0.090 [0.083, 0.097]		0.052 [0.048, 0.056]		0.040 [0.037, 0.043]	

*p < 0.05.

**Table 4:** The impact of contextual factors on the effectiveness of timeout in advantage status.

Variables		Time windows					
		Short-term		Mid-term		Long-term	
		EMM [95%CI]	*p* (ES)	EMM [95%CI]	*p*	EMM [95%CI]	p
Game period	Regular	−0.081 [−0.108, −0.055]	0.274 (small)	−0.035 [−0.057, −0.013]	0.737 (small)	−0.015 [−0.033, 0.002]	0.443 (small)
	Last 5 min	−0.109 [−0.159, −0.058]		−0.028 [−0.071, 0.015]		−0.001 [−0.039, 0.037]	
Opposition quality	High-quality	−0.101 [−0.139, −0.063]	0.014* (moderate)	−0.038 [−0.071, −0.006]	0.663 (small)	−0.002 [−0.030, 0.025]	0.517 (trivial)
	Medium-quality	−0.053 [−0.084, −0.022]		−0.024 [−0.050, 0.003]		−0.018 [−0.042, 0.006]	
	Low-quality	−0.131 [−0.387, −0.075]		−0.032 [−0.080, 0.016]		−0.004 [−0.045, 0.038]	
Score status	Winning	−0.057 [−0.095, -0.020]	0.003* (moderate)	−0.006 [−0.037, 0.025]	0.156 (trivial)	−0.004 [−0.030, 0.022]	0.937 (trivial)
	Balanced	−0.068 [−0.103, −0.034]		−0.030 [−0.059, −0.001]		−0.009 [−0.032, 0.015]	
	Losing	−0.160 [−0.216, −0.104]		−0.058 [−0.107, −0.010]		−0.011 [−0.055, 0.033]	

*p < 0.05.

Table 5 presents the results of pairwise comparisons from the linear mixed-effects model. Timeout effects across all time windows (long-, mid-, and short-term) were superior during regular game time compared to the last 5 min (*p* < 0.001, Small). When opposition quality was medium, mid-term momentum was significantly higher than against high-quality opponents (*p* = 0.002, Trivial). Long-term effects were superior in balanced and winning states compared with losing states (*p* = 0.021 and 0.046, Small). Under disadvantage status, the long-term effect was superior in balanced and winning status compared to losing status (*p* < 0.001 and *p* = 0.011, Small). Both short-term and long-term effect was superior compared with facing low-quality opponents than high-quality (*p* = 0.025 and *p* = 0.012, Trivial). Under advantage status, short-term effects were weaker against medium-quality opponents compared to low-quality (*p* = 0.033, Large), and worse under losing status compared to balanced and winning (*p* = 0.005 and *p* = 0.007, Very Large to Large).

**Table 5:** Comparison of effectiveness of timeout under different contextual factors.

Status	Time windows	Variable [compare]	Difference	SE	*p*	Effect size
All	Short-term	Game period [last 5 min—regular]	−0.020	0.004	<0.001	Small
	Mid-term	Game period [last 5 min—regular]	−0.010	0.003	<0.001	Small
	Mid-term	Opposition quality [high quality—medium quality]	−0.006	0.002	0.002	Small
	Long-term	Game period [last 5 min—regular]	−0.007	0.002	<0.001	Small
	Long-term	Score status [balanced—losing]	−0.004	0.001	0.021	Small
	Long-term	Score status [losing—winning]	0.004	0.002	0.046	Small
Disadvantage	Short-term	Opposition quality [high quality—low quality]	−0.011	0.004	0.025	Trivial
	Long-term	Opposition quality [high quality—low quality]	−0.006	0.002	0.012	Trivial
	Long-term	Score status [balanced—losing]	−0.005	0.002	<0.001	Trivial
	Long-term	Score status [losing—winning]	0.005	0.002	0.011	Trivial
Advantage	Short-term	Opposition quality [low quality—medium quality]	−0.078	0.029	0.033	Large
	Short-term	Score status [balanced—losing]	0.092	0.025	0.005	Very large
	Short-term	Score status [losing—winning]	−0.103	0.030	0.007	Large

## 4 Discussion

This study evaluated the impact of timeouts on momentum in basketball games. Compared with previous studies that mainly relied on static indicators such as scores and possessions to evaluate timeout effects, we introduced momentum across three time windows—short-term (48 s), mid-term (96 s), and long-term (144 s), to dynamically and continuously assess shifts in momentum following a timeout across different time scales. Furthermore, we distinguished timeout events occurring under disadvantageous and advantageous team states, and examined the moderating role of contextual factors on different types of timeouts. Results showed that timeouts generally increased team momentum, particularly when the team was in a disadvantage status. However, momentum declined following timeouts during advantage status. Additionally, the effect of timeouts on game momentum was moderated by contextual factors, especially in mid- and long-term windows. Although some analyses yielded effect sizes categorized as Trivial or Small, in the context of high-level competition, even subtle changes may exert meaningful impacts on the game. These findings offer new perspectives for understanding the dynamic effects of timeouts and highlight the importance for coaches to consider both game status and contextual conditions in timeout decisions.

Timeouts are recognized by coaches as a means to disrupt opponents’ dominant performance during actual games. Theoretically, existing studies generally suggest that timeouts function by suppressing opponents’ scoring runs, facilitating tactical adjustments, and altering game tempo (Mace et al., 1992; Sampaio et al., 2013). Early studies provided preliminary support for this view. Mace proposed the reinforcement mechanism, suggesting that timeouts reduce reinforcement cues for opponents while creating new reinforcement opportunities for the team (Mace et al., 1992). Subsequent findings supported this hypothesis, Gómez found that both offensive and defensive performance improved after timeouts, suggesting that timeouts function by both reducing opponents’ reinforcers and enhancing those of the team (Gómez et al., 2011). In fact, due to the ambiguous nature of momentum and the difficulty in isolating its effects, the impact of timeouts on momentum has not been thoroughly and systematically explored.

Based on the momentum framework proposed by Qiu et al. (2024), we found that teams calling timeouts exhibited momentum increases in the short-, mid-, and long-term windows, thereby supporting the effectiveness of timeouts. Our findings first support the effectiveness of timeouts, as the calling team exhibited momentum increases across short-, mid-, and long-term windows. This result partially echoes previous possession-based studies, which revealed that timeouts could improve team performance in the subsequent possessions (Gómez et al., 2011; Sampaio et al., 2013). Our study, however, reveals that the effects of timeouts can extend to influencing the course of the game over longer time scales. The two approaches reveal the mechanisms of timeouts from different perspectives and are therefore complementary. As momentum is calculated by changes in point differential over time, it reflects not just scoring runs but also the dynamic shifts in both offensive and defensive performance over a given period. Considering the stochastic nature of basketball games, timeouts serve to disrupt the opponent’s advantageous state and either restore randomness or shift the game into the team’s favorable momentum phase (Hastie, 1999). Thus, our findings suggest that timeouts can positively influence game dynamics across multiple timeframes—not only by immediately disrupting opponents but also by facilitating longer-term tactical improvements. These findings imply that timeouts should be regarded not merely as emergency interventions, but also as strategic tools for mid- and long-term game adjustment and tempo regulation, offering support for their role in shaping game dynamics. However, these results are not fully consistent with studies based on NBA samples, which often report weak or negligible timeout effects (Permutt, 2011; Assis et al., 2021). This discrepancy is primarily attributable to differences in outcome measures and in the operational definitions of momentum. The league context is another source of discrepancy: the NBA includes mandatory timeouts and commercial considerations, and differences in timeout usage patterns, timing, and coaching characteristics between the NBA and CBA further influence research outcomes. In addition, Assis et al. argued that post-timeout performance recovery is largely a result of score regression to the mean rather than the intervention effect of the timeout itself (Assis et al., 2021). By contrast, in our analysis, we incorporated pre-timeout states (advantaged/disadvantaged) to minimize the risk of misinterpreting natural regression as a timeout effect.

This study found that 77.5% of timeouts were called when teams were in a disadvantaged state, consistent with previous research (Gómez et al., 2011; Gutiérrez-Aguilar et al., 2016; Prieto et al., 2016; Lloveras and Vollmer, 2022; Lovato and Barreira, 2025). This suggests that timeouts are typically used by coaches as a tactical tool to disrupt opponents, adjust strategies, and stabilize team mentality. Sports psychologists note that teams under pressure often experience elevated anxiety, cognitive overload, diminished focus, and reduced tactical execution (Taylor and Wilson, 2005). Therefore, timeouts serve both tactical and psychological functions, allowing coaches to use verbal encouragement to help players regain focus and confidence. Under disadvantageous conditions, teams showed significant momentum gains across all time windows, supporting the “rebound mechanism” of timeouts. Notably, the mid-term timeout effect under disadvantage was unaffected by contextual variables, indicating greater situational robustness than other timeframes.

Conversely, less than 1% of timeouts were called under advantageous conditions, which is unsurprising. From a coaching perspective, calling a timeout during a favorable state is often viewed as a high-risk, low-reward decision. On the one hand, a timeout may interrupt the positive feedback loop that the team is forming (such as consecutive scoring, quick passing, and coordinated defense) (Weimer et al., 2023). On the other, they may offer the opponent chances to adjust tactically and mentally, altering the game momentum (Halldorsson, 2016; Lovato and Barreira, 2025). To our knowledge, this is the first study to explore timeouts under absolute advantage. Our findings indicate that calling timeouts in advantageous states may be associated with momentum decline, a trend consistent with the “perturbation hypothesis” from dynamic systems theory, where external interventions can disrupt optimal system performance (Davids et al., 2008). Given the small sample size (n = 35), the generalizability of these findings is highly limited. Nonetheless, this exploration opens a window for future research on coaches’ timeout decisions in advantageous states and suggests that such timeouts should be used with caution, as coaches must balance rhythm and tactical fluidity to avoid overcontrol.

The impact of timeouts on momentum varies across game contexts, suggesting that the intervention mechanism is influenced by multiple external factors. From a temporal perspective, basketball is inherently time-dependent, with identical actions yielding different effects at different time points (Gómez et al., 2017; Sampaio et al., 2010). We observed that timeouts are more effective during regular periods than in the last 5 min. This may be partly attributed to the slower pace and increased scoring unpredictability during the closing moments of the game (Bar-Eli et al., 1996; De Saá Guerra et al., 2013), as well as to players being more susceptible to external pressure and environmental distractions, which compromise decision-making quality and lead to errors or irrational shot selections (Schweickle et al., 2021). Allgrunn’s study further supports this, showing that calling a timeout before a potential game-tying or go-ahead possession actually reduces scoring success (Allgrunn et al., 2024). However, our findings may also have been influenced by the specific short-timeout rules in the CBA. Short timeouts are restricted to the final 2 min of the game and are considerably shorter in duration than regular timeouts, which may limit the effectiveness of coaches’ in-game interventions.

Our findings also support the notion of the Interacting Performances Theory, which posits that opponent quality not only affects match outcomes but also alters tactical strategies and technical performances (Dong et al., 2021; O’Donoghue, 2009). We observed that timeouts were more effective when facing lower-quality opponents, which is an expected outcome. In basketball, team–opponent interactions are inevitable, and the stronger side typically exhibits superior tactical execution and control over game tempo (Zhang et al., 2019). Therefore, timeouts tend to be less effective against stronger opponents, as intervention strategies are more easily counteracted. Particularly in advantageous situations, timeouts against high-quality opponents are more likely to diminish the team’s momentum, further supporting the notion that unnecessary timeouts may offer strategic opportunities to opponents (Halldorsson, 2016; Lovato and Barreira, 2025). Score status also moderates the effect of timeouts, with timeouts being less effective when the team is in a losing position, suggesting that coaches should enhance situational awareness and avoid delayed interventions in already adverse scenarios. Notably, our findings revealed that the sensitivity of timeout effects to contextual factors differs across time windows. Specifically, medium- and long-term effects are more influenced by contextual variables than short-term effects. This indicates that medium- and long-term effects are more vulnerable to external situational disruptions, and their effectiveness depends more heavily on game context and conditions, whereas short-term effects tend to be more stable and context-resilient.

Despite its contributions, this study has several limitations that should be acknowledged. First, regarding the application of momentum, the three temporal windows were nested, which may have resulted in high correlations among the measures and reduced statistical independence. Moreover, fixed time windows may obscure the influence of specific critical possessions following a timeout, leading to conclusions that are biased toward averaged effects. Compared with possession-based approaches to analyzing timeout effects, this method may be limited in capturing tactical execution, for instance, by not directly reflecting the effectiveness of the first offensive or defensive possession after a timeout. Future research may consider using non-nested dynamic windows (e.g., sliding windows) or hybrid models combining temporal and possession-based units, thereby more comprehensively capturing the dual effects of timeouts at both immediate and sustained levels. The definitions of advantaged/disadvantaged states were also relatively strict, approximating an “idealized” scenario that effectively reduced random fluctuations in conclusions but limited coverage of more complex mixed situations. Second, the study used an observational design and primarily employed t-tests, Wilcoxon signed-rank tests, and mixed-effects models to explore momentum changes before and after timeouts. While these methods help identify associations, it has weaker capabilities in causal inference. Future studies could adopt more causally robust statistical approaches, such as propensity score matching, inverse probability weighting, or structural causal models, to further examine the intervention effect of timeouts on momentum. In addition, this study did not differentiate between long and short timeouts, nor did it examine the specific intervention strategies used by coaching staff during timeouts (e.g., tactical adjustments, motivational communication, or player substitutions), which may have led to variability in the content and quality of interventions. Ignoring these differences may cause the results to reflect the average effect of timeouts rather than specific mechanisms and outcomes; future studies should consider timeout types and intervention strategies to provide more practical guidance. Lastly, the sample was drawn exclusively from the CBA, which, despite being a top-tier professional league, differs from European and American leagues in game characteristics and coaching practices, thereby limiting the generalizability of the findings. Future studies should compare leagues and competition levels to enhance the practical relevance of the results.

## 5 Conclusion

This study found that timeouts generally help enhance team momentum, with more pronounced effects under disadvantageous conditions. Conversely, calling a timeout during advantageous situations may reduce momentum, suggesting that coaches should exercise greater caution when leading. Additionally, the effects of timeouts are moderated by various contextual factors, especially in the mid- and long-term windows, while short-term effects appear more contextually stable. Coaching staff should better recognize situational characteristics and intervention timing in real-time decision-making to maximize the value and effectiveness of timeouts. These findings offer new insights into the dynamic regulatory function of timeouts in basketball and provide practical guidance for improving coaching decisions and in-game tactical adjustments.

## Data Availability

The raw data supporting the conclusions of this article will be made available by the authors, without undue reservation.
